# Adolescents exposed to discrimination: are they more prone to excessive internet use?

**DOI:** 10.1186/s12887-020-02241-3

**Published:** 2020-08-25

**Authors:** Laura Bitto Urbanova, Jana Holubcikova, Andrea Madarasova Geckova, Jitse P. van Dijk, Sijmen A. Reijneveld

**Affiliations:** 1 Department of Health Psychology and Research Methodology, Faculty of Medicine, P. J. Safarik University, Trieda SNP 1, 040 11 Kosice, Slovakia; 2 Graduate School Kosice Institute for Society and Health, Faculty of Medicine, P. J. Safarik University, Kosice, Slovakia; 3 Department of Community and Occupational Medicine, University Medical Center Groningen, University of Groningen, Groningen, the Netherlands; 4 Olomouc University Society and Health Institute, Palacky University Olomouc, Olomouc, Czech Republic

**Keywords:** Discrimination, Excessive internet use, Gender, Adolescents

## Abstract

**Background:**

The Internet may serve as a suitable environment for discriminated adolescents as they may consider the online space as the place where they have possibility to build social ties they are missing in their offline life or manage their self-presentation. Therefore, our aim was to explore the association between different types of discrimination by peers (because of gender, physical appearance, culture/skin color/language, unfavorable family situation) and excessive Internet use (EIU), and whether gender moderates this association.

**Methods:**

We used data from a representative sample of 6,462 Slovak adolescents (mean age: 13.00, 49.6% boys) from the HBSC study conducted in 2018. Data were collected through online self-reported questionnaires. We assessed the association between various types of discrimination by peers and EIU using linear regression, and the role of gender as potential moderator.

**Results:**

Discrimination because of physical appearance was most prevalent (18.0%). Adolescents exposed to discrimination by peers reported higher levels of EIU. We found an interaction of gender on the association of discrimination because unfavorable family situation with EIU. Boys who experienced this type of discrimination were at higher risk of EIU compared to girls.

**Conclusion:**

Discriminated adolescents are more likely to use the Internet excessively, with some associations being stronger for boys than for girls. Prevention strategies focused on raising adolescent awareness of the risks and benefits of the Internet should target discriminated adolescents, especially boys, as they seem to be the vulnerable group.

## Background

The Internet can provide a comfortable environment for adolescents experiencing difficulties in their life [1]. Excessive use of it, however, can have a detrimental impact on different life domains. Excessive Internet use (EIU) can cause problems of a physical (headache, backache, dry eyes, neck pain, insomnia), psychological (increase of depressive symptoms, anxiety, stress) and social nature (social isolation) [2–5]. According to Smahel et al. [6] Internet use can be considered as excessive if children are unable to control their online activities what can cause negative consequence on their normal life. Griffiths [7] discerns the following basic components in his model of Internet addictive behavior: salience, tolerance, mood change, withdrawal symptoms, interpersonal and intrapersonal conflict, and relapse, focused on the negative consequences and also on the way people feel about those experiences. However, there is lack of agreement on the extent to which a situation regards excessive Internet use only or it can be described as an addiction. Previous research showed an increase in the prevalence of risky Internet users from 13.3 to 17.6% in the years from 2009/2010 to 2011/2012 [8]. Regarding the Slovak context, in 2017 94% of Slovak people under the 25 years used Internet on a daily basis [9] and almost 20% of Slovak adolescents reported that they more easily talk about their inner feelings and fears in the online world [10]. This preference for online communication can next lead to EIU. The results of the EU Kids Online study (2020) showed that about 6% of Slovak adolescents met 1–2 criteria of EIU and 2% reported to meet 3–4 criteria of EIU [11]. Evidence is scarce on the early stages of Internet addiction and its impact on adolescents’ lives.

Discrimination may be one of the factors leading to EIU. Discrimination may be invoked by a variety of characteristics, such as gender, body image, race, disability, socioeconomic status or religion [12–17]. Previous research has shown frequent and multiple exposure to discrimination to be associated with poor mental and physical health [18, 19]. Discriminated adolescents also felt less socially competent in peer relationships [20], which can result in social isolation [21, 22]. Previous research has also shown several other factors to be related to EIU. According to Kim, Larose and Peng [23], people who have problems maintaining healthy relationships in the offline space, who are shyer, have less confidence in themselves [24] and a higher level of loneliness [25], may find it difficult to control their online activities, which can then lead to EIU. Furthermore, results of the EU Kids Online study (2011) showed that about 10% of the discriminated or psychologically disadvantaged children, aged between 9 and 16 years old, reported being bullied online [26]. However, evidence is lacking on the association between discrimination in the offline space and Internet use, as most studies regard discrimination via the Internet and its impact on an adolescent’s life [27, 28].

Previous research has suggested there are gender differences for several types of discrimination, which may also affect associations with EIU. An increase in symptoms of depression, anxiety or psychological distress was associated with boys experiencing racial discrimination and young women experiencing gender discrimination [13, 14, 29]. Regarding discrimination because of physical appearance, overweight girls reported more exposure to stigmatization and bullying [12]. However, evidence is scarce on the association between different types of discrimination and EIU, and whether gender moderates this association.

Therefore, our aim was to assess the association between various types of discrimination by peers, i.e. because of gender, physical appearance, culture/skin color/language and unfavorable family situation, and excessive Internet use, and whether gender moderates this association. We expect that adolescents exposed to some type of discrimination are at higher risk of EIU.

## Methods

### Sample and procedure

We used data from the Health Behaviour in School-aged Children (HBSC) study conducted in 2018 in Slovakia. To obtain a representative sample, a two-step sampling procedure was used. First, 140 larger and smaller schools located in rural as well as in urban areas from all regions of Slovakia were randomly selected from a list of all eligible schools in Slovakia and asked to participate in the study. The school response rate (RR) was 77.9%. Second, we obtained data from 8405 adolescents from 11 to 15 years old (RR = 60.0%; mean age = 13.43; 50.9% boys) by using online self-reported questionnaires.

The sample designed for our study comprised the adolescents who answered the questions that measured the different types of discrimination and EIU. This regarded 6462 adolescents (mean age = 13.00, 49.6% boys), the other 1943 were excluded because of missing values. Non-response per items varied between 13,1% and 19.9%. Higher proportions of missing responses could be caused by the fact that the examined items were situated in the second part of the questionnaire. Moreover, adolescents might find it difficult to talk openly about their experiences with discrimination, and the level of their Internet use.

Our study was approved by the Ethics Committee of the Medical Faculty at the P.J. Safarik University in Kosice (16 N/2017). We informed parents about the study via the school administration, and they could opt out if they disagreed with their child’s participation. Participation in the study was fully voluntary and confidential with no explicit incentives provided for participation.

### Measures

We measured the level *of excessive Internet use* in adolescents using the EIU scale [30]. This scale consists of five items with 4-point Likert-type responses, covering the five dimensions of behavioural addiction [7]. These dimensions are: salience (I have gone without eating and sleeping because of the Internet); withdrawal symptoms (I have felt bothered when I cannot be on the Internet); tolerance (I have caught myself surfing when I am not really interested); relapse (I have tried unsuccessfully to spend less time on the Internet); and conflict (I have spent less time than I should with either family, friends, or doing schoolwork because of the time I spend on the Internet). Responses that adolescents could choose were (1) never/almost never, (2) not very often, (3) fairly often, (4) very often. The total EIU score regards the sum score of all items (in this sample M = 7.68, SD = 2.92; and Cronbach’s alpha 0.78).

*Discrimination* by peers was measured by a question set on discrimination because of *gender* (How often pupils at school treat you unfairly or negatively because you are a boy or a girl?); physical *appearance* (How often do pupils at school treat you unfairly or negatively because of your figure or appearance?); *culture/SC/L* (How often do pupils at school treat you unfairly or negatively because you are using different language or because of different color of your skin?); and *unfavorable family situation* (How often do pupils at school treat you unfairly or negatively because bad situation at home (money, illness or something else)?). Responses were: (1) never, (2) hardly ever, (3) sometimes, (4) often, (5) very often. This item was dichotomized per domain as: discriminated (sometimes, often, very often), not discriminated (never, hardly ever).

Family affluence was measured using the Family Affluence Scale. We computed the sum score, which we converted to a score ranging from 0 to 1. We then created tertile categories of low (0 to 0.333), medium (0.334 to 0.666) and high (0.667 to 1) socio-economic position.

We used gender as a potential moderating variable.

### Statistical analyses

First, we assessed the background characteristics, the mean value of EIU, and the prevalence of different types of discrimination, overall and by gender; we examined gender differences by using chi-square tests. Second, we assessed the association between various types of discrimination by peers and EIU (transformed to z-score), crude and adjusted for gender and age, using ordinary linear regression. Third, we analyzed the degree to which gender modified the effect of different types of discrimination by adding their interactions to the regression model. We analyzed our data using IBM SPSS statistics 20.0 for Windows.

## Results

### Description of the sample

Descriptive statistics of the sample are presented in Table 1. Less than 20% of our sample reported being discriminated by their classmates for any reason. The most prevalent was discrimination because of physical appearance. Boys seemed to be more exposed to discrimination because of culture/SC/L and unfavorable family situation. In reverse, the prevalence of discrimination because of physical appearance was significantly higher in girls. Of adolescents, 25.7% reported being exposed to at least one type of discrimination, this prevalence being slightly higher for girls than for boys (*p* < 0.001).

**Table 1:** Background characteristics of the sample overall and stratified by gender (6,462 Slovak adolescents aged 11–15 years old, HBSC Study 2018)

	Total sample *N* = 6462 (100%)	Boys *N* = 3203 (49.6%)	Girls *N* = 3259 (50.4%)	Difference Boys/Girls *p*-value
Discriminated because of				
Gender (n, %)	891 (13.8)	430 (13.4)	461 (14.1)	ns
Physical appearance (n, %)	1164 (18.0)	512 (16.0)	652 (20.0)	***
Culture/ SC/L (n, %)	316 (4.9)	184 (5.7)	132 (4.1)	**
Unfavorable family situation (n, %)	315 (4.9)	175 (5.5)	140 (4.3)	*
EIU (range 5 (low) – 20 (high)) (Mean, SD, t-test)	7.68 (2.9)	7.70 (3.1)	7.65 (2.7)	ns

Missing values N (%): discrimination because of gender 1101 (13.1%), discrimination because of physical appearance 1160 (13.8%), discrimination because of culture/ SC/L 1136 (13.5%), discrimination because of unfavorable family situation 1148 (13.7%), excessive Internet use 1673 (19.9%)

*ns* not significant, *EIU* excessive Internet use

### Associations between different types of discrimination and EIU

Discriminated adolescents reported higher levels of EIU (Model 1, Table 2). Regarding the crude model, we found no gender differences in reporting EIU (Model 1, Table 2). Adjustment for gender and age hardly changed the association between different types of discrimination and EIU (Model 2, Table 2). Additional adjustment for family affluence hardly changed findings (findings not shown).

**Table 2:** Association of excessive Internet use with discrimination by peers because of gender, physical appearance, culture/SC/L or unfavourable family situation: crude betas, betas adjusted for gender, age and interaction terms of discrimination per type with gender; results of linear regression analysis leading to standardized regression coefficients (β) (6462 Slovak adolescents aged 11–15 years old, HBSC Study 2018)

	Model 1			Model 2			Model 3		
	β	B (95%CI)	p	β	B (95%CI)	p	β	B (95%CI)	p
**Discrimination by peers because of gender**	0.15	0.44 (0.37;0.51)	***	0.15	0.44 (0.37;0.51)	***	0.11	0.63 (0.41;0.85)	***
Gender (male vs. female)				0.00	0.03 (−0.05;0.05)	ns	0.03	0.02 (− 0.03;0.07)	ns
Age				0.14	0.10 (0.09;0.12)	***	0.10	0.10 (0.09;0.12)	***
Discrimination because of gender x male gender							0.07	−0.13 (−0.26;0.011)	ns
**Discrimination by peers because of physical appearance**	0.16	0.42 (0.35;0.48)	***	0.16	0.41 (0.35;0.47)	***	0.18	0.47 (0.269;0.67)	***
Gender (male vs. female)				−0.01	−0.01 (−0.06;0.04)	ns	−0.00	−0.01 (− 0.06;0.05)	ns
Age				0.13	0.10 (0.08;0.12)	***	0.13	0.10 (0.08;0.12)	***
Discrimination because of physical appearance x male gender							−0.02	−0.04 (−0.16;0.09)	ns
**Discrimination by peers because of culture/SC/L**	0.15	0.71 (0.60;0.82)	***	0.15	0.71 (0.60;0.82)	***	0.19	0.86 (0.53;1.20)	***
Gender (male vs. female)				0.01	0.02 (−0.03;0.06)	ns	0.01	0.02 (−0.03;0.07)	ns
Age				0.14	0.10 (0.09;0.12)	***	0.14	0.10 (0.09;0.12)	***
Discrimination because of culture/SC/L x male gender							−0.04	−0.11 (−0.33;0.12)	ns
**Discrim. by peers because of unfavorable family situation**	0.15	0.67 (0.56;0.78)	***	0.14	0.65 (0.54;0.76)	***	0.26	1.23 (0.89;1.57)	***
Gender (male vs. female)				0.01	0.01 (−0.04;0.06)	ns	0.02	0.03 (−0.02;0.08)	ns
Age				0.13	0.10 (0.08;0.12)	***	0.13	0.10 (0.08;0.12)	***
Discrimination because of unfavorable family situation x male gender							−0.13	−0.40 (−0.62;-0.17)	**

Model 1 Crude effect of discrimination on EIU

Model 2 The effect of discrimination on EIU adjusted for gender and age

Model 3 With added interaction of discrimination with gender on EIU

*p < 0.05, ** p < 0.01, *** p < 0.001; ns not significant

### Modification by gender of the associations of discrimination and EIU

We found a statistically significant interaction of gender with only one type of discrimination, i.e. because of unfavorable family situation, regarding the association between discrimination and EIU (Model 3, Table 2). The interaction of gender with discrimination because of gender, physical appearance and culture/SC/L with EIU remained non-significant (Model 3, Table 2).

We repeated the analyses with a stratification for gender regarding the association of discrimination because of unfavorable family situation with EIU. These associations were significant among both boys and girls, but the effect of discrimination on EIU differed between genders. Boys who were exposed to discrimination because of unfavorable family situation reported higher levels of EIU than girls.

## Discussion

We assessed the associations between different types of discrimination by peers with EIU in Slovak adolescents and a potential moderating effect of gender on this association. Our results showed that almost 20% of the adolescents questioned were exposed to discrimination, most frequently to discrimination because of physical appearance. Adolescents who experienced discrimination tended to report higher levels of EIU. The effect of discrimination by peers because of unfavorable family situation on EIU seemed to be stronger in the case of boys.

We found discrimination by peers to be associated with a higher level of EIU among Slovak adolescents. This contrasts to previous studies conducted on this topic, as we found that discriminated adolescents are not only more likely to experience online risky behavior, such as sexting or cyberbullying [31, 32], but they are also at higher risk of becoming excessive Internet users. Our results can be explained in several ways. First, discrimination by peers can leave discriminated adolescents feeling less socially competent, subsequently socially isolated, with lack of social support [20, 21]. In today’s society the Internet can be considered as an alternative tool for building social ties that adolescents are missing in their offline life [33, 34]. According to Davis’s cognitive–behavioral model of problematic Internet use (PIU), one of the major causes of PIU are maladaptive cognitions users have about themselves. The online space can be seen by discriminated adolescents as a place where they have the possibility to control their self-presentation and gain more positive reactions from other users [35, 36]. This kind of positive experience can easily lead to more frequent use of the Internet, which can then lead to EIU. Second, Internet use can be seen by discriminated children as a coping mechanism for their mental health problems. Previous research has shown an association between discrimination and an increase in depression symptoms or anxiety [37–41]. Discriminated adolescents can then consider the Internet as a place where they can find social support from people who are experiencing similar life conditions or from experts, such as psychologists, with whom they may communicate more easily about their situation than in face-to-face interaction.

We found that boys exposed to discrimination by peers because of unfavorable family situation were at higher risk of EIU compared with girls. Previous research on the association between discrimination and EIU moderated by gender is completely lacking, as most recent studies have been dedicated to online discrimination. We can explain these findings in several ways. First, girls tend to report a higher level of conscientiousness and agreeableness already in earlier adolescence compared to boys [42]. Those personality traits are fundamental for the creation of positive interpersonal relationships specific for girls [43]. Furthermore, there is an increase in girls’ self-disclosure during pre– and early adolescence, while in boys the increase of self-disclosure typically occurs 2 years later [44], which can cause a tendency for adolescent boys to be shyer at a similar age. Adolescent girls are also more likely to have a higher level of social support from their peers, which helps them handle negative feelings caused by discrimination. Second, boys try to respond to discrimination using their personal capacities, which can lead to the adoption of different types of unhealthy behaviors, such as frequent smoking [45], substance use [46] or EIU. According to Lenhart [47], boys are more likely to make friends online by playing online games or using social media. Regarding the types of discrimination, previous research has shown that boys are more sensitive to unfavorable family situations [48]. For example, boys who experience some kind of unfavorable family situation, such as a parental divorce, tend to report more problematic behaviors, such as delinquency and substance use [49]. However, there is a lack of evidence on the association between different types of discrimination and EIU.

### Strengths and limitations

The major strength of this study is its large and nationally representative study sample of adolescents. We collected our data through the EIU scale, which is a validated method used previously in various reports [6, 30]. However, some limitations should also be mentioned, too. First, the cross-sectional design of our study prevents the exploration of causal pathways the used variables. Next, we used self-report questionnaires, meaning that respondents’ answers may be affected by social desirability. Adolescents may find it difficult to disclose they are discriminated against; so discrimination might be somewhat under reported, which can then result in a low proportion of different types of discrimination. To get as close to reality as possible, we used online anonymous questionnaires, which probably reduced this effect. Furthermore, in our study we focused only on one direction of the association between different types of discrimination by peers and EIU. Exploration of reverse pathway could provide also interesting findings, as spending of much time online can reduce the number of social interactions which adolescents have in the offline world. This can lead to social isolation, subsequently.

Finally, selection bias may have occurred as only 76.9% of respondents were included into the analysis. The included respondents regarded less boys, younger respondents and respondents from low family affluence background than the excluded respondents. From previous studies [11, 50, 51] we might expect that boys and respondents from lower SES are at higher risk of EIU, while younger respondents are at lower risk of EIU. This could have affected the strengths of the associations as studied, but only to a limited degree given the relatively small percentage that was excluded.

### Implications

Our findings that discrimination is associated with EIU implies a need for interventions focused on reducing the social exclusion of discriminated adolescents resulting from peer relationships. Such interventions should target boys in particular, as they seem to be the more vulnerable group. Moreover, our results also show a need for preventive strategies focused on raising adolescents’ knowledge about the risks and benefits associated with Internet use. In our study we explored only EIU in general, but a deeper analysis of specific online activities may bring useful information for developing preventive interventions. Moreover, further research should explore the context of demographic characteristics; such as socioeconomic status, school grade or place of living. Finally, longitudinal research may help to explore the causal pathways between discrimination and EIU.

## Conclusion

We found an association between different types of discrimination by peers (because of gender, physical appearance, culture/SC/L and unfavorable family situation) and EIU; gender moderated this association. Adolescents exposed to discrimination reported a higher level of EIU. Boys who experienced discrimination because of the unfavorable family situation were more vulnerable to becoming excessive Internet users than girls. Preventive strategies should focus on raising adolescents’ awareness about the benefits and risks of Internet use, especially among discriminated adolescents, as they seem to be a vulnerable group.

## Data Availability

The data used in this study are available from the corresponding author on reasonable request.

## Abbreviations

EIU: Excessive Internet use
HBC study: Health Behavior in School- aged children study
